# The Importance of Surface-Binding Site towards Starch-Adsorptivity Level in *α*-Amylase: A Review on Structural Point of View

**DOI:** 10.1155/2017/4086845

**Published:** 2017-12-05

**Authors:** Umi Baroroh, Muhammad Yusuf, Saadah Diana Rachman, Safri Ishmayana, Mas Rizky A. A. Syamsunarno, Jutti Levita, Toto Subroto

**Affiliations:** ^1^Master of Biotechnology Program, Postgraduate School, Universitas Padjadjaran, Jl. Dipati Ukur 35, Bandung, West Java, Indonesia; ^2^Department of Chemistry, Faculty of Mathematics and Natural Sciences, Universitas Padjadjaran, Jl. Raya Bandung-Sumedang Km 21, Jatinangor, Sumedang, West Java 45363, Indonesia; ^3^Research Center of Molecular Biotechnology and Bioinformatics, Universitas Padjadjaran, Jl. Singaperbangsa 2, Bandung, West Java 40133, Indonesia; ^4^Department of Pharmacology and Clinical Pharmacy, Faculty of Pharmacy, Universitas Padjadjaran, Jl. Raya Bandung-Sumedang Km 21, Jatinangor, Sumedang, West Java 45363, Indonesia

## Abstract

Starch is a polymeric carbohydrate composed of glucose. As a source of energy, starch can be degraded by various amylolytic enzymes, including *α*-amylase. In a large-scale industry, starch processing cost is still expensive due to the requirement of high temperature during the gelatinization step. Therefore, *α*-amylase with raw starch digesting ability could decrease the energy cost by avoiding the high gelatinization temperature. It is known that the carbohydrate-binding module (CBM) and the surface-binding site (SBS) of *α*-amylase could facilitate the substrate binding to the enzyme's active site to enhance the starch digestion. These sites are a noncatalytic module, which could interact with a lengthy substrate such as insoluble starch. The major interaction between these sites and the substrate is the CH/pi-stacking interaction with the glucose ring. Several mutation studies on the* Halothermothrix orenii*, SusG* Bacteroides thetaiotamicron*,* Barley*,* Aspergillus niger*, and* Saccharomycopsis fibuligera α*-amylases have revealed that the stacking interaction through the aromatic residues at the SBS is essential to the starch adsorption. In this review, the SBS in various *α*-amylases is also presented. Therefore, based on the structural point of view, SBS is suggested as an essential site in *α*-amylase to increase its catalytic activity, especially towards the insoluble starch.

## 1. Introduction

Starch is the most abundant form of storage of many economically important crops such as wheat, rice, maize, tapioca, and potato [1, 2]. Starch-containing crop is an essential constituent of the human diet, and a large proportion of the food consumed by the world's population originates from them. Starch is harvested and used as its original form or chemically or enzymatically processed into a variety of different products, for example, starch hydrolysates, glucose syrups, fructose, starch or maltodextrin derivatives, or cyclodextrins [1].

Degradation of starch into a variety of different products is performed by amylolytic enzymes, such as *α*-amylase, glucoamylase, *β*-amylase, isoamylase, pullulanase, exo-1,4-*α*-D-glucanase, *α*-D-glycosidase, and cyclomaltodextrin-D-glucotransferase [3].

The amylases are multidomain proteins. Interestingly, about 10% of amylases contain a distinct noncatalytic module that is known to facilitate binding and degradation of raw starch [4]. Initially, only two types of starch-binding domains (SBDs) were recognized: either very frequent C-terminal SBD or very scarcely occurring N-terminal SBD [5]. However, sometimes the substrate also binds to one or more surface regions called surface-binding site (SBS) [6]. In starch-based industry, *α*-amylase is used to break down the starch granules, which are densely packed in a polycrystalline state by inter- and intramolecular bonds. Starch granules are insoluble in cold water and often resistant to chemicals and enzymes [7]. A gelatinization step at a high temperature (105°C) would help to open the crystalline structure of starch. Hence it is easier to be digested by the enzyme [8]. Nevertheless, this process requires high energy, thus resulting in high cost of production [9]. Therefore, starch processing in lower temperature is more preferred [8, 10, 11]. The ability of the amylolytic enzyme to hydrolyze the raw starch was related to the level of starch-adsorptivity properties [11].

Amylolytic enzymes with raw starch digesting ability may contain SBD and/or SBS. Hence, in this review, we focus on the importance of starch-binding particularly SBSs. From a structural point of view, there are five examples of *α*-amylases, with or without SBS, which can be used to review the following aspects: (1) the most significant factor in starch-binding, (2) the type of interactions that influence the binding of these proteins to the substrate in the noncatalytic module, and (3) the reason of low substrate adsorptivity to the protein despite having high amylolytic activities.

## 2. Carbohydrate-Binding Module in Amylolytic Enzymes

In general, carbohydrate-active enzymes that degrade or modify polysaccharides bind to the substrate on the carbohydrate-binding site situated outside of the active-site area. These additional binding sites can be found on the carbohydrate-binding modules (CBMs) or the surface-binding sites (SBSs) [18].

Cellulose-binding domain (CBD) was originally defined as noncatalytic polysaccharide-recognizing module of glycoside hydrolases (GHs). This module binds ligand such as cellulose and the other carbohydrates. Afterward, the term of carbohydrate-binding module (CBM) was used to reflect the diverse ligand specificity of these modules [19]. Many CBMs have been identified experimentally, and hundreds of CBMs were further identified based on the amino acid similarity [20]. There are currently 81 defined families of CBMs (http://www.cazy.org/Carbohydrate-Binding-Modules.html), and these CBMs showed substantial variation in ligand specificity (Table 1).

**Figure 1:**
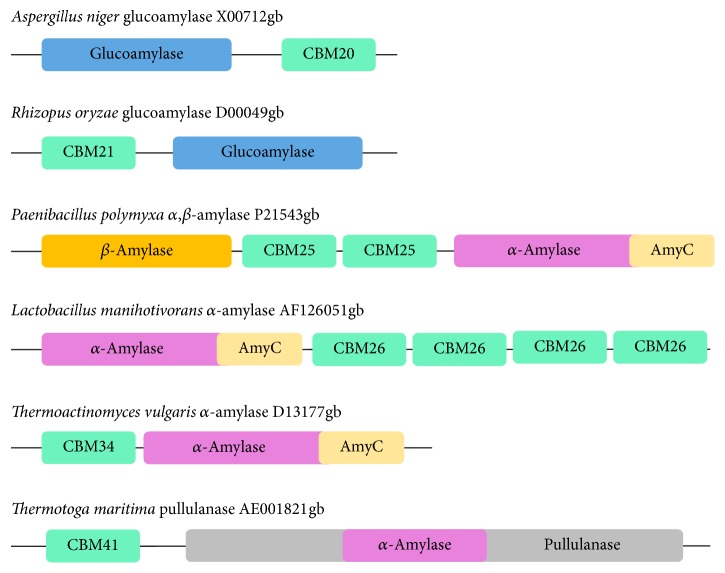
The classical architectures of starch-binding domains (SBDs), which are CBM20, CBM21, CBM25, CBM26, CBM34, and CBM41. SBDs are found at the N- or C-termini of the catalytic domain and are shown in turquoise colored boxes. The catalytic domains (CD) of glucoamylase, *β*-amylase, *α*-amylase, and pullulanase are highlighted in blue, yellow, purple, and grey colors, respectively. Accession numbers are retrieved from GenBank (adapted from Rodríguez-Sanoja et al. [12]).

CBM in starch-hydrolyzing enzymes is called starch-binding domain (SBD). SBDs have been identified in *α*-amylase, *β*-amylase, maltotetraohydrolase, maltopentaohydrolase, maltogenic *α*-amylase, cyclodextrin glucanotransferase (CGTase), acarbose transferase, and glucoamylase [14]. The illustrative view of classical SBD architectures is shown in Figure 1.

In general, the roles of CBM in the associated catalytic modules are in the proximity effect, the targeting function, and the disruptive function. Through this sugar-binding activity, the concentrated substrate on the surface of the protein can enhance the speed of degradation of polysaccharide [20].

**Figure 2:**
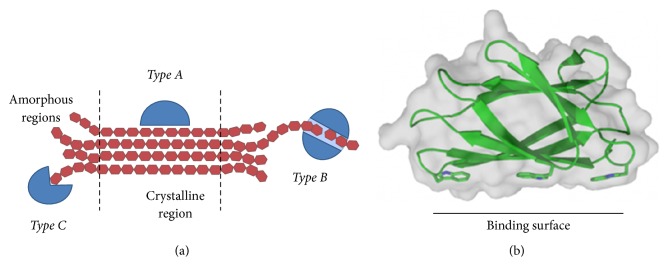
(a) Types A, B, and C of CBM bind to polysaccharides in a different region. (b) Type A of CBM2 from* Pyrococcus furiosus* (PDB ID code 2CRW [13]) shows that aromatic residues form a planar binding surface (adapted from [14]).

There are three types of CBM regarding the form of substrates, types A, B, and C (Figure 2(a)). Type A binds to the crystalline surfaces of cellulose and chitin (e.g., CBM1, CBM2, CBM3, CBM5, and CBM10 families). Their binding sites are composed of many aromatic residues, creating a flat platform to bind to the planar polycrystalline chitin or cellulose surface (Figure 2(b)). Type B, which is currently the most abundant form of CBMs, binds to the internal glycan chains (*endo*-type). The type B binding sites formed as extended grooves or clefts comprised binding subsites to accommodate longer sugar chains (four or more monosaccharide units), for example, CBM6, CBM36, and CBM60. Lastly, type C binds to the termini of glycans (reducing/nonreducing ends,* exo*-type). This site appears as a small pocket which can recognize a short sugar ligand containing one to three monosaccharide units (e.g., CBM9, CBM13, CBM32, CBM47, CBM66, and CBM67 families) [12].

However, the noncatalytic carbohydrate-binding module does not only exist as CBM. A growing number of structural studies on various GHs have also revealed the presence of carbohydrates bound to one or more noncatalytic surface regions of the catalytic module. Carbohydrate-binding in such surface-binding sites, that is, SBSs, occurs in a fixed position relative to the catalytic site. It is different from the noncatalytic binding in CBMs, which are usually attached to the flexible loop structure [6].

Starch granules possess crystalline and amorphous forms which are rigid and difficult to be degraded. Hence the strategy to enhance the catalytic efficiency is through the incorporation the SBSs in various enzymes. However, SBSs are restricted not only to starch-active enzymes, but also in other GHs with different specificities, belonging to several GH families, and originating from mammal, plant, archaea, fungi, and bacteria. Several functions of SBS in GHs are (1) targeting towards its substrate, (2) assisting catalysis by loading substrates into the active-site pocket, (3) disrupting of the structure of substrates to facilitate catalysis, (4) keeping a substrate chain in contact with the enzyme for subsequent reactions, (5) allosteric activation of the enzyme, (6) retention and passing on the reaction products, and (7) anchoring the GH to the cell wall of the host microorganism [22–24].

CBM and SBS are crucial for starch binding. The differences between these binding sites are located on the architecture of binding. SBS is usually formed by aromatic residue on the surface of the enzyme. The importance of SBS to the starch adsorptivity in various *α*-amylases will be discussed below.

## 3. Surface-Binding Site in *α*-Amylase

### 3.1. *Halothermothrix orenii α*-Amylase B

*Halothermothrix orenii* is an anaerobic, halophilic, thermophilic, Gram-negative bacterium isolated from the sediment layer of a Tunisian salt lake in the Sahara desert. This bacterium experiences variations of salt concentration and temperature over time. The optimum pH, temperature, and salt (NaCl) concentration for the growth of* H. orenii* cells are 6.5–7.0, 60°C, and 1.7 M, respectively [25].

*H. orenii *produces two *α*-amylases, AmyA and AmyB. AmyB has an additional N-terminal domain (N domain) that forms a large groove, the N–C groove, located around 30 Å away from the active site. This N domain is important for hydrolyzing the insoluble starch by improving the binding ability of AmyB to the insoluble substrate [15].

**Figure 3:**
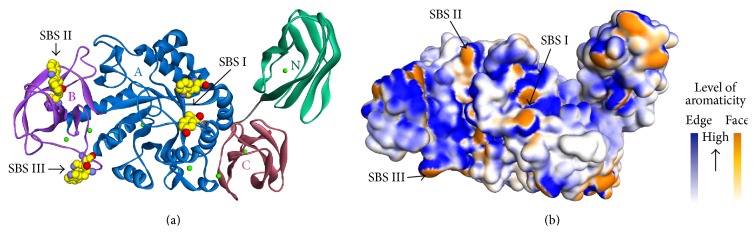
The overall structure of AmyB (PDB ID code 3BCD [15]). (a) Ribbon structure of AmyB with domains N, A, B, and C colored in green, blue, violet, and pink. Three SBSs are highlighted by a black arrow with a yellow sphere as the critical residues for binding. The eight metal ions are colored in green and purple balls. (b) Molecular surface of AmyB structure based on the aromaticity of residues. The face-side of aromatic residues forms three SBSs on the surface of AmyB.

AmyB consists of three domains, A, B, and C domain (Figure 3(a)). The A domain features the typical (*β*/*α*) 8 TIM barrel. The active site is located at the C-terminal end of the TIM barrel, composed of D350, E380, and D447 as the catalytic residues. The B domain is located between the strand *β*3 and the helix *α*3 of the A domain. The interaction between A and B domain is also stabilized by the presence of a metal triad (Ca^2+^–Na^+^–Ca^2+^). Lastly, the C domain folds as a C-terminal eight-stranded *β* sandwich, following the *α*/*β*-barrel. The N domain folds into a nine-stranded immunoglobulin-like *β* sandwich of fibronectin III type. Although the A domain forms extensive interdomain interactions with B and C domain, it has limited interactions with the N domain [15].

Two structures of AmyB have been deposited in the Protein Data Bank. The first structure was complexed with acarbose (AmyB_acr_), whereas the second one was complexed with maltoheptaose/cyclodextrin (AmyB_mal7–acx_). Three SBSs were found in the crystal structures: two SBSs in the acarbose-bound complex and another SBS in the maltoheptaose/cyclodextrin-bound complex [15]. Several aromatic residues were found on the surface of this structure (Figure 3(b)).

**Figure 4:**
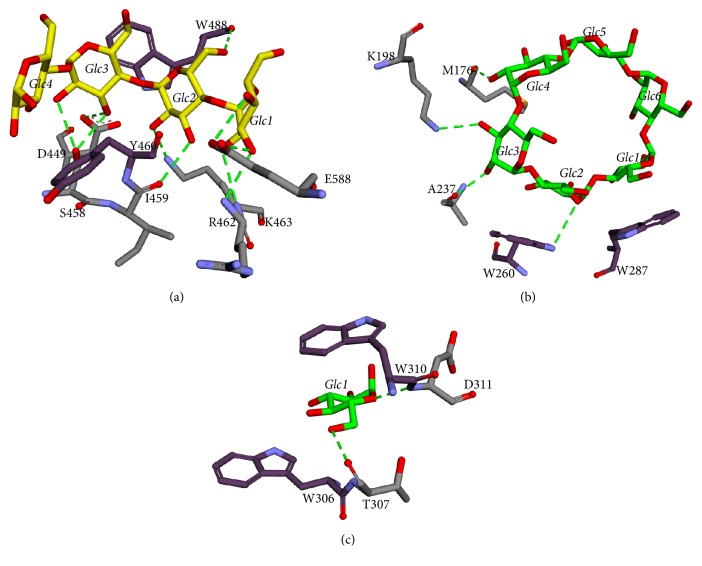
The molecular interactions around (a) SBS I, (b) SBS II, and (c) SBS III. The tetrasaccharide, *β*-cyclodextrin, and glucose are represented in yellow and green colored sticks, respectively. Aromatic residues and the other amino acids around the substrate that formed hydrogen bonds are shown in dark purple and grey colored sticks, respectively. A hydrogen bond is depicted in green dashed line.

A tetrasaccharide was present in the SBS I site of AmyB_acr_ and AmyB_mal7–acx_. Two aromatic residues, W488 and Y460, formed CH/pi-stacking interactions with Glc3 and Glc4, respectively. There are also ten potential hydrogen bonds, that is, E588 with O4 and O3 of Glc1, K463 with O2 of Glc2, R462 with O3 of Glc1, I459 with O3 of Glc2, S458 with O2 and O3 of Glc3, D449 with O3 of Glc3, and W488 with O6 of Glc2 (Figure 4(a)). In SBS II, a *β*-cyclodextrin binds to the AmyB_mal7–acx_. Two tryptophans were found on this site, W287 and W260 that formed CH/pi-stacking interactions with Glc1 and Glc2, respectively. There are also four potential hydrogen bonds, W260 with O5 of Glc2, A237 with O2 of Glc3, K198 with O3 of Glc3, and M176 with O2 of Glc4 (Figure 4(b)). In SBS III, a glucose binds to the AmyB_acr_. Two tryptophans, W310 and W306, formed stacking interactions with the glucose. There are also two potential hydrogen bonds, T307 with O6 of Glc1 and D311 with O5 of Glc1 (Figure 4(c)).

Interestingly, the N and C grooves also contained aromatic residues that could interact favorably with carbohydrates. The deletion of N domain decreased the starch degradation performance of AmyB as compared to the full-length sequence. This result suggests the vital role of N domain to sequester and to render the natural starch to be more accessible for further processing and hydrolysis [15].

### 3.2. SusG* Bacteroides thetaiotaomicron α*-Amylase

SusG (starch utilization system G)* Bacteroides thetaiotaomicron α*-amylase is part of a large protein complex on the outer surface of the bacterial cell. It plays a significant role in carbohydrate acquisition by the animal gut microbiota. SusG is expressed concurrently with Sus-CDEF on the outer surface of the cell and is required for cell growth on starch [16].

**Figure 5:**
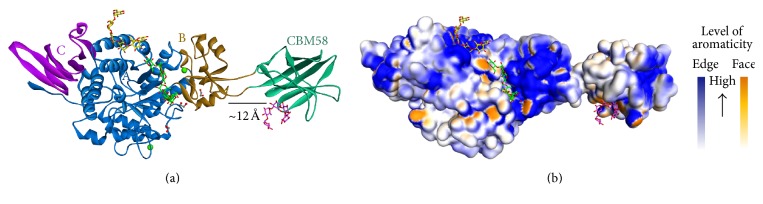
The overall structure of SusG (PDB ID code 3K8L). (a) The ribbon structure of SusG. The A, B, and C domain and CBM58 are colored in blue, brown, purple, and green, respectively. The metal ions are displayed as a green sphere, and those of ethylene glycol molecules are in grey. The maltoheptaose is represented differently based on its location at the active site, the secondary starch-binding site (SBS), and CBM58 (green, yellow, and pink colored sticks, resp.). (b) The molecular surface of SusG structure based on the edge- and face-side of aromatic residues [16].

The structure of SusG is composed of A, B, and C domain that share structural features with the other *α*-amylases (Figure 5(a)). The A domain contains the catalytic site, with the B domain inserted between *β*3 and *α*3 of the A domain. The B domain contributes to the size and accessibility of the active site, whereas the C domain is a standard feature of many GH13 family enzymes. SusG displays an unusual extended shape, ~12 Å in length, due to the insertion of a CBM58 that protrudes from the B domain. CBM58 makes no direct contact with the A, B, and C domain and it is linked to the core of amylase structure by two short linkers, located 12 Å away from the B domain. Naturally, these linkers are not flexible and do not directly interact with each other, either the core domains or the CBM58. They have a few potentials of interdomain water-mediated hydrogen bonds. SusG also has a secondary starch-binding site in the A domain, which is similar to the SBS [16]. Based on the aromaticity of residues on the surface of SusG, it is shown that the aromatic residues are spread around the active site and starch-binding site or SBS (Figure 5(b)).

**Figure 6:**
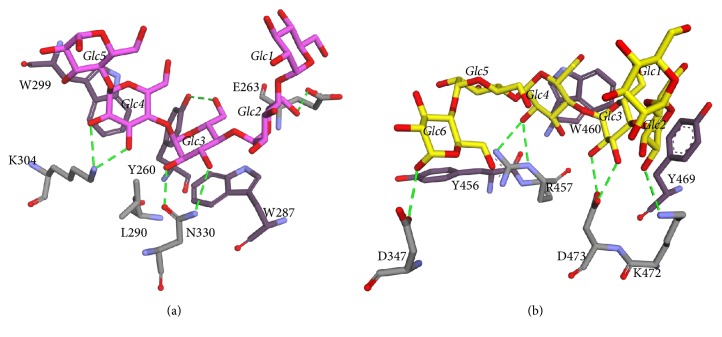
Molecular interaction around the substrate-binding site. (a) The binding of maltopentaose (pink) to the CBM58. (b) The binding of maltoheptaose (yellow) to the SBS. Aromatic residues are visualized in a darker color. Hydrogen bonds denoted by green dashed lines.

Five glucose residues of maltoheptaose are well ordered at the CBM58. In this binding site, there are two CH/pi-stacking interactions between W287 and W299 to Glc3 and Glc4, respectively. The L290 formed hydrophobic interaction with both tryptophans. W299 has potentially formed a T-shape stacking interaction with Y260. Besides, there are also six potential hydrogen bonds: E263 with O6 of Glc2, N330 with O2 and O3 of Glc3, Y260 with O6 of Glc3, and K304 with O2 and O3 of Glc4 (Figure 6(a)). The pattern of starch binding at the CBM58 is comprised of hydrophobic interactions with the additional hydrogen bonding to the 2′ and 3′ hydroxyl groups of the adjacent glucose residues. This pattern is a conserved feature of many starch-binding CBMs [26]. In addition, this binding pattern is also observed in SusD [27], barley, and pancreatic *α*-amylases that bind raw starch on the surface of the catalytic domain [28, 29].

In addition to CBM, the SBS in SusG also has a similar characteristic. It contains tryptophan and tyrosine in the binding site. The Y469 formed CH/pi stacking with Glc2 and W460 formed stacking with Glc4. It is also noted that six potential hydrogen bonds were formed: D437 with O1 of Glc6, R457 with O2 of Glc4, D473 with O2 and O3 of Glc3, and K472 with O2 of Glc2 (Figure 6(b)).

Some mutation studies of this enzyme revealed that stacking interaction is essential to the starch-binding. The first mutant of SusG lacking CBM58, namely, mCBM58, was generated by deleting residues 210–339 and inserting the five residues loop GSPTG, similar to that observed in the* H. orenii *amylase A, a close structural homolog of SusG without CBM58. The second mutant, namely, mSURF, was constructed by mutating the surface-binding site (W460A/Y469A/D473V) to test the importance of these residues to the starch-binding capability. The mCBM58, mSURF, and WT SusG enzymes were tested for their enzymatic activity using p-nitrophenyl-maltopentaose (PNP-G5). Their catalytic turnover rates were identical. The enzymes were then tested for their ability to degrade the soluble starch, amylopectin, pullulan, and insoluble cornstarch. For each substrate, the activity of WT SusG was used as the positive control (100%), and the mCBM58 and mSURF mutant enzymes were compared to the wild-type. The mCBM58 showed the highest activity to all substrates except for the insoluble cornstarch in which the activity was remarkably decreased up to 71%, whereas mSURF had the lowest activity for all substrates. Interestingly, its activity on the insoluble corn starch was also decreased up to 56%. Therefore, both the CBM58 and the SBS are required for the optimal degradation of insoluble corn starch [16].

### 3.3. *Barley α*-Amylase Isozyme 1

Barley *α*-amylase isozymes (AMY1 and AMY2) of subfamily GH13_6 [2] are among the first carbohydrate-active enzymes identified with the SBS [24, 30]. Although the SBS was first discovered in AMY2, the characterization of functional properties of these SBSs was performed on the AMY1. The reason was due to the higher yields of recombinant AMY1 produced by* Pichia pastoris, *which was about 60-fold higher than AMY2 [31]. Moreover, another preliminary work indicated that the starch binding to SBS2 in AMY2 is weaker than in AMY1. This finding was also confirmed by its crystal structure [32].

**Figure 7:**
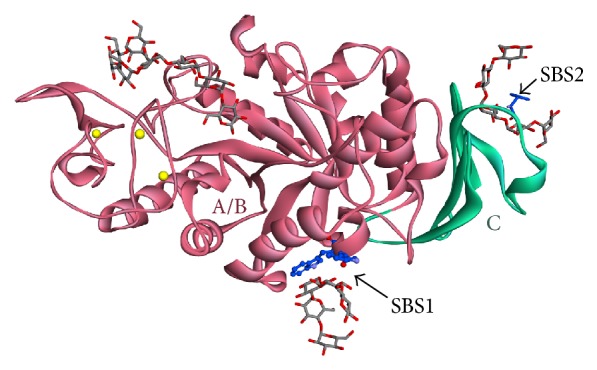
The ribbon structure of AMY1. The A/B and C domains are colored in pink and green, respectively. The metal ion is colored in yellow spheres. The maltopentaose and maltohexaose (grey sticks) bind to the SBS and active site, respectively.

Similar to the other *α*-amylases, AMY1 has A, B, and C domain. The A/B domain consists of the catalytic domain, while the C domain is a common feature of many GH13 family enzymes. It is worth noting that CBM is not present in AMY1. However, two SBSs exist: SBS1 and SBS2 (Figure 7).

**Figure 8:**
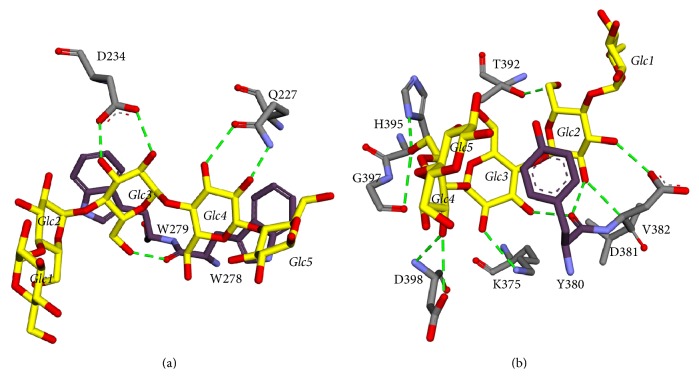
The 3D interaction of maltoheptaose (yellow) bound to the (a) SBS1 and (b) SBS2. Hydrogen bonds and aromatic residues are denoted by green dashed lines and dark violet sticks, respectively.

In SBS1, two aromatic residues interact with the maltopentaose, that is, W278 and W279. These tryptophans formed CH/pi-stacking interactions with Glc3 and Glc4, respectively. There are also five potential hydrogen bonds around this site, that is, Q227 with O2 and O3 of Glc4, the backbone of W278 with O6 of Glc3, and D234 with O2 and O3 of Glc3 (Figure 8(a)).

In SBS2, there is an aromatic residue which formed CH/pi-stacking interaction with maltopentaose, that is, Y380. There are also ten potential hydrogen bonds around this site, that is, V382 with O2 of Glc2, D381 with O3 of Glc2, Y380 with O2 of Glc2 and with O3 of Glc3, K375 with O2 of Glc3, D398 with O3 of Glc4, G397 with O6 of Glc4, H395 with O6 of Glc4, and T392 with O6 of Glc2 (Figure 8(b)).

SBS1 is known as starch granule binding site, and SBS2 is known as a pair of sugar tongs [18]. Nielsen and colleagues have performed the mutation of Y380A in the SBS2 [24]. As a result, its activity decreased about tenfold (*Kd* = 1.4 mg/mL) as compared to the wild-type AMY1. The mutant retained less than half of the activity to release the soluble reducing sugars from starch granules. Furthermore, it was noticed that these effects were more prominent for single or double SBS1 alanine mutants of W278 and W279. The complete loss of affinity for barley starch granules (*Kd* > 100 mg/mL) resulted when both of SBSs were modified using triple mutations W278A/W279A/Y380A. This mutant retained only 0.2% of the wild-type hydrolytic activity towards barley starch granules [24]. In contrast, both affinity and rates of hydrolysis were increased roughly tenfold when a starch-binding domain of the CBM20 family from* Aspergillus niger* glucoamylase was fused with the C-terminal of AMY1 [31].

The architecture of both SBSs corresponds to their distinct roles. A binding platform in SBS1 comprised two tryptophans, whereas the “pair of sugar tongs” in SBS2 formed by Y380 and H395, which are positioned to accommodate an individual chain of the substrate. SBS1 is suggested as the initial site for AMY1 attachment to the starch granule surface. SBS2 is a supporting site for substrate binding near the *α*-1,6 branch point. Thus, it feeds a linear segment of the amylopectin into the active site, which is unable to accommodate branches near the point of hydrolysis. Once AMY1 inserts the starch granule surface, the role of SBS1 in the catalytic activity would be over. In contrast, SBS2 is continuously isolating the individual chains to be delivered to the active site [33].

### 3.4. *Aspergillus niger α*-Amylase

*Aspergillus niger α*-amylase is classified as a member of GH family 13 among the 109 GH families that are currently identified. Its sequence is 100% identical to the* A. oryzae* homolog. Its crystal structure with a resolution of 3.0 Å was reported in 1984 (PDB ID code 2TAA, [34]) and known as TAKA-amylase [17].

**Figure 9:**
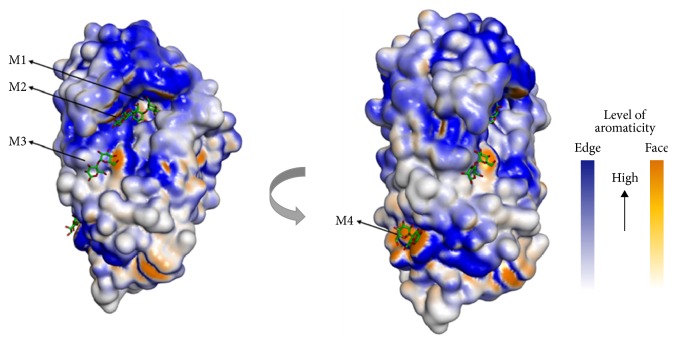
The molecular surface of* A. niger α*-amylase based on the aromaticity of amino acid. Four maltoses (substrates) bound to the surface, rich in aromatic residues.

**Figure 10:**
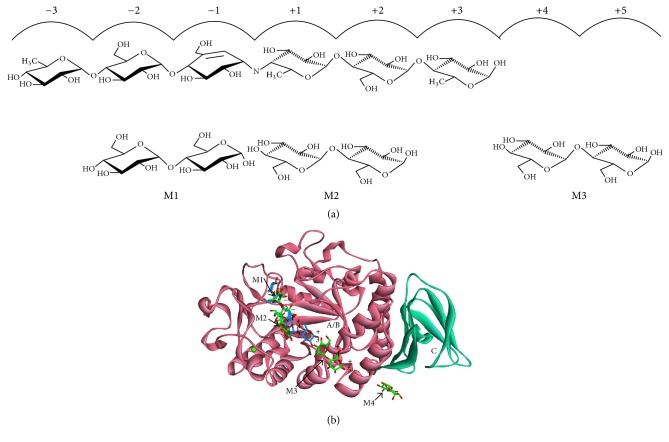
The overall structure of* A. niger α*-amylase and its ligand. (a) The comparison of subsites for acarbose and maltose binding (adapted from Vujicic-Zagar and Dijkstra [17]). (b) The structure of* A. niger α*-amylase. The A/B and C domains are colored in pink and turquoise, respectively. Acarbose and maltose are represented by blue and green colored sticks, respectively. A cofactor calcium ion is visualized by a green sphere.

*A. niger α*-amylase in complex with maltose, the simplest substrate of this enzyme, has been published with a PDB ID code 2GVY at 1.6 Å resolution. This structure consists of four maltose molecules bound on the protein surface composed of aromatic residues (Figure 9). It is found that the two maltoses were in unusual position when compared to the acarbose in TAKA-amylase (PDB ID code 7TAA). The structure of this enzyme has a typical *α*-amylase structure with A, B, and C domain: A/B domain as a catalytic module and C domain as a standard feature like the other *α*-amylases (Figure 10). Three molecules of maltose were found in the active site in subsite −1 and −2, +1 and +2, and +4 and +5. Another maltose was found in 20 Å distance from subsite +5. This site was later known as the SBS, which is located on a loop between A and C domain. Its function is to bind the polysaccharide chain extending from the active site. The plasticity of the active-site groove in the proximity to the catalytic center might be substantial for both formations of the productive substrate-enzyme complex as well as for the release of the product from the +1 to +*n* subsites [17].

**Figure 11:**
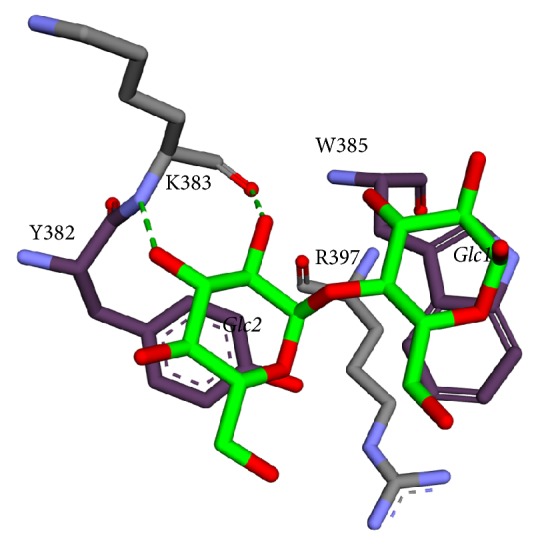
Molecular interactions around the SBS of* A. niger α*-amylase. Maltose is represented in green stick, aromatic residue in dark purple stick, and hydrogen bond in green dashed lines.

The M4 molecule (maltose) formed hydrophobic stacking interactions with Y382 and W385 which are located on the loop connecting the last helix of the TIM barrel and the first-strand of the C domain (Figure 11). These sites were involved in the binding of a long carbohydrate chain extending from the active site. In addition, R397 was found to stabilize the two aromatic residues with hydrophobic interaction.

### 3.5. *Saccharomycopsis fibuligera α*-Amylase

*S. fibuligera* is a food-borne yeast that is widely used in the production of rice or cassava-based fermented food [35]. The yeast, in combination with* Saccharomyces cerevisiae* or* Zymomonas mobilis*, has been used in the production of ethanol using cassava starch as the starting material [36].

One of the best strains of this yeast,* S. fibuligera* R64, produces two amylolytic enzymes: *α*-amylase (Sfamy) and glucoamylase (GluR) [37]. Sfamy has an optimum temperature of 50°C and is active in a broad pH range with an optimum pH of 5.0. The digestion of native Sfamy with trypsin resulted in two major fragments with apparent molecular masses of 39 kDa (p39) and 10 kDa (p10), respectively. The two fragments represent the N- and C-terminal domains of the *α*-amylase. According to Matsuura et al. [34], the N-terminal domain of *α*-amylase consists of the integrated A and B domains, in which the active site is located. The C-terminal domain consists of C domain, in which its function in Sfamy is not yet established [38].

Hasan et al. [38] reported that Sfamy has no starch binding as compared to the GluR, which has the adsorption level of 90%, 80%, 25%, and 20% to the maize, tapioca, sago, and potato starches, respectively [38].

**Figure 12:**
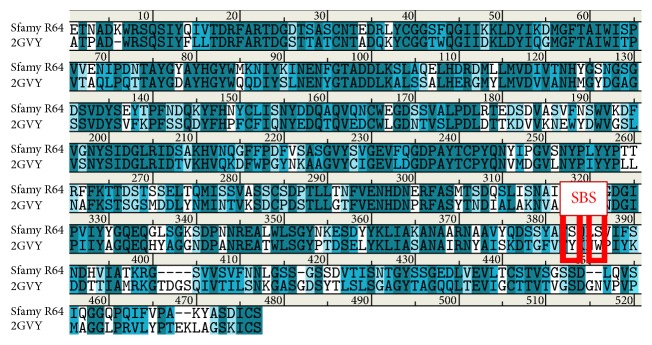
Sequence alignment between Sfamy R64 and* A. niger α*-amylase. The SBS is highlighted by red colored box.

A computational study on the differences between Sfamy and* A. niger α*-amylase was conducted as an effort to understand the low adsorptivity of Sfamy on the raw starch [39]. The sequence and homology model of Sfamy were aligned to that of* A. niger α*-amylase (PDB ID code 2GVY) [17]. The sequence of Sfamy was retrieved from NCBI with accession code HQ172905.1 [40]. As a result, these sequences shared 54% identity and 71% homology. At the SBS region, Sfamy has two serines, while* A. niger α*-amylase has two aromatic residues (Figure 12). This difference was suggested as the reason of the low adsorptivity of Sfamy on the raw starch. Although the two serines could form hydrogen bonds with the substrate, which usually occurred in the starch-binding process, they might not be strong enough to hold the substrate on the enzyme's surface.

Furthermore, molecular dynamics simulations were performed on the structure of Sfamy and* A. niger α*-amylase to investigate their time-dependent structural behavior of substrate binding. The substrate in Sfamy was not consistently bound to the SBS region, while that in* A. niger α*-amylase was stable over the simulation. Interestingly, a double mutant of S383Y/S386W of Sfamy showed a comparable substrate-binding activity to that of* A. niger*'s. These introduced aromatic residues formed CH/pi-stacking interaction with the substrate [39].

In general, the interaction between CBM and carbohydrate is weak (Ka affinities in mM^−1^ to *μ*M^−1^ range), hence making the interaction easily reversible. Once catalysis has been completed at the particular site, there is “recycling” of the appended enzyme to bind to a new region on the substrate [41]. It is suggested that the most important driving force mediating the protein-carbohydrate interactions is the position and orientation of aromatic residues within the SBS, such as tyrosine, tryptophan, or phenylalanine. These planar residues formed essential hydrophobic stacking interactions with the planar face of sugar rings. Moreover, it was noted that weak intermolecular electrostatic interactions, which occurred between CH and pi electrons in the planar ring systems, contributed around 1.5 to 2.5 kcal/mol energy to the binding reaction [42]. However, the geometric features of the interaction are not strictly unique. From the point of view of the protein structure, different architectures of the binding sites can be described, depending on the number and relative location of aromatic residues [41]. In Protein Data Bank, more than 90 of nonredundant 3D structures of CBD show carbohydrate aromatic stacking. This type of interaction has resulted in the improvement of protein modeling strategies, especially those that are of a low similarity, by introducing a “hydrophilic aromatic residue” parameter as a restriction for structural modeling [43].

## 4. Conclusion

Starch binding in *α*-amylases, with or without SBS, is shown to be influenced by the presence of CH/pi-stacking interaction. This interaction occurs between aromatic residues (tyrosine, tryptophan, and sometimes phenylalanine) and the partial positively charged hydrogen atom of the substrate. These aromatic residues should have a specific topology to bind well to the substrate. Also, their conformations have to be stable (e.g., stabilized by hydrophobic interaction around aromatic residue). The CBM or SBS does not significantly influence the catalytic activities towards the short chain polysaccharides, but they are essential to hydrolyze the long or insoluble starch (raw starch). Therefore, the presence of SBS should be considered as the critical aspect of improving the starch adsorptivity of *α*-amylase.

## Figures and Tables

**Table 1:** CBM classification based on ligand specificity (http://www.cazypedia.org, taken from Barchiesi et al. [21]).

Ligand	CBM family
Cellulose	CBM1, CBM2, CBM3, CBM4, CBM6, CBM8, CBM9, CBM10, CBM16, CBM17, CBM28, CBM30, CBM37, CBM44, CBM46, CBM49, CBM59, CBM63, CBM64, CBM65, CBM73, CBM76, CBM78, CBM80, CBM81

Xylan	CBM2, CBM4, CBM6, CBM9, CBM13, CBM15, CBM22, CBM31, CBM35, CBM36, CBM37, CBM44, CBM54, CBM59, CBM60, CBM64, CBM72

Plant cell wall, other (e.g., *β*-glucans, porphyrans, pectins, mannans, gluco- and galacturonans)	CBM4, CBM6, CBM11, CBM13, CBM16, CBM22, CBM23, CBM27, CBM28, CBM29, CBM32, CBM35, CBM39, CBM42, CBM43, CBM52, CBM56, CBM59, CBM61, CBM62, CBM67

Chitin	CBM1, CBM2, CBM5, CBM6, CBM12, CBM13, CBM14, 16 CBM18, CBM19, CBM50, CBM54, CBM55, CBM73

*α*-Glucans (starch/glycogen, mutant)	CBM20, CBM21, CBM25, CBM26, CBM34, CBM41, CBM45, CBM48, CBM53, CBM58, CBM68, CBM69, CBM74

Mammalian glycans	CBM32, CBM40, CBM47, CBM51, CBM57

Other	Bacterial cell wall sugar: CBM35, CBM39, CBM50 Fructans: CBM38, CBM66 Yeast cell wall glucans: CBM54
